# Metabolomics Study of Resina Draconis on Myocardial Ischemia Rats Using Ultraperformance Liquid Chromatography/Quadrupole Time-of-Flight Mass Spectrometry Combined with Pattern Recognition Methods and Metabolic Pathway Analysis

**DOI:** 10.1155/2013/438680

**Published:** 2013-05-26

**Authors:** Yunpeng Qi, Haiwei Gu, Yunlong Song, Xin Dong, Aijun Liu, Ziyang Lou, Guorong Fan, Yifeng Chai

**Affiliations:** ^1^Department of Pharmaceutical Analysis, School of Pharmacy, Second Military Medical University, 325 Guohe Road, Shanghai 200433, China; ^2^Shanghai Key Laboratory for Pharmaceutical Metabolite Research, 325 Guohe Road, Shanghai 200433, China; ^3^Shanghai Research Centre for Drug (Chinese Materia Medica) Metabolism, 325 Guohe Road, Shanghai 200433, China; ^4^Jiangxi Key Laboratory for Mass Spectrometry and Instrumentation, East China Institute of Technology, Nanchang, Jiangxi 330013, China; ^5^Department of Anesthesiology & Pain Medicine, University of Washington, Seattle, WA 98195, USA; ^6^Department of Pharmacology, School of Pharmacy, Second Military Medical University, 325 Guohe Road, Shanghai 200433, China

## Abstract

Resina draconis (bright red resin isolated from *Dracaena cochinchinensis*, RD) has been clinically used for treatment of myocardial ischemia (MI) for many years. However, the mechanisms of its pharmacological action on MI are still poorly understood. This study aimed to characterize the plasma metabolic profiles of MI and investigate the mechanisms of RD on MI using ultraperformance liquid chromatography/quadrupole time-of-flight mass spectrometry-based metabolomics combined with pattern recognition methods and metabolic pathway analysis. Twenty metabolite markers characterizing metabolic profile of MI were revealed, which were mainly involved in aminoacyl-tRNA biosynthesis, phenylalanine, tyrosine, and tryptophan biosynthesis, vascular smooth muscle contraction, sphingolipid metabolism, and so forth. After RD treatment, however, levels of seven MI metabolite markers, including phytosphingosine, sphinganine, acetylcarnitine, cGMP, cAMP, L-tyrosine, and L-valine, were turned over, indicating that RD is likely to alleviate MI through regulating the disturbed vascular smooth muscle contraction, sphingolipid metabolism, phenylalanine metabolism, and BCAA metabolism. To our best knowledge, this is the first comprehensive study to investigate the mechanisms of RD for treating MI, from a metabolomics point of view. Our findings are very valuable to gain a better understanding of MI metabolic profiles and provide novel insights for exploring the mechanisms of RD on MI.

## 1. Introduction 

Myocardial ischemia (MI) is characterized by an imbalance between the supply and demand of myocardial oxygen, causing cardiac dysfunction, arrhythmias, myocardial infarction, and sudden death [1, 2]. Despite the advances in drug development, MI has become a leading cause of death globally [3, 4]. According to an investigation carried out by the World Health Organization, an estimate of 17.5 million people died from cardiovascular disease, of which 7.6 million were due to MI (http://www.who.int/cardiovascular_diseases/en/).

Resina draconis (also called “dragon's blood”), a bright red resin isolated from *Dracaena cochinchinensis*, is one of the renowned traditional medicines and has been used as a “panacea of blood activation” in China for many years. It has been clinically used for the treatment of cerebral arterial thrombosis, ischemic heart disease and blood stasis syndrome, and so forth [5, 6]. Specifically, total flavones in resina draconis has shown positive effects on myocardial ischemia animals [7, 8]. However, the mechanisms of its pharmacological action on MI are still poorly understood, mostly due to its complex components.

Metabolomics aims to reveal various metabolic characteristics of external or internal perturbations to biological systems by profiling low-molecular-weight metabolites in biosamples [9–13]. Metabolic changes are the most proximal reporters of alterations in the body in response to a disease process or drug therapy. Metabolic profiling of MI and its drug treatment are hence important in understanding MI pathology and therapeutic mechanisms of drugs including resina draconis.

This study aimed to characterize the plasma metabolic profiles of MI rats and investigate the mechanisms of resina draconis on MI using metabolomics approaches. Among the multiple analytical platforms commonly applied in metabolomics, ultra-performance liquid chromatography/quadrupole time-of-flight mass spectrometry (UPLC-Q-TOF-MS) offers improved resolution, greater sensitivity, and higher speed and has been regarded as one of the premier tools for the identification and quantitation of metabolites in complex biological samples [14]. In order to process complicated data of metabolite profiles and extract useful information, multivariate statistical analysis and pattern recognition methods were used to handle UPLC-Q-TOF-MS data [15], after which bioinformatics analyses based on MBRole and IPA were performed leading to further identification of biological association networks and unveiling the possible mechanisms of resina draconis on MI.

## 2. Experimental

### 2.1. Materials and Animals

HPLC grade acetonitrile was purchased from JT Baker (NJ, USA). Distilled water was purified in-house using a Milli-Q20 system from Millipore (MA, USA). Total flavone extract of resina draconis was provided by Yulin Pharmacy Company (Xishuangbanna, China). Isosorbide dinitrate, a vasodilator that has been proven to relax vascular smooth muscles resulting in a decrease in both venous return and arterial blood pressure [16, 17], was used as the positive control drug and purchased from Forward Co., LTD (Shanghai, China). Commercial standards (including phytosphingosine, L-phenylalanine, L-tyrosine, L-tryptophan, methionine, and L-valine) were purchased from Sigma/Aldrich (MO, USA). Methanol was bought from Sinopharm Chemical Reagent Co. (Shanghai, China). Carboxymethyl cellulose- (CMC-) Na was purchased from Shanghai Genebase Gene-Tech Co. (Shanghai, China). The assay kits for lactate dehydrogenase (LDH) and creatine kinases (CK) were purchased from Shanghai Fosun Long March Medical Science Co. (Shanghai, China). Sprague-Dawley rats (180~210 g, male) were purchased from the Sippr-Bk Lab Animal Ltd. Co. (Shanghai, China) and fed with certified standard diet and tap water. Temperature and humidity were set at 21–23°C and 40–60%, respectively. A 12 h light/dark cycle was used.

### 2.2. *In Vivo* Experiments Protocol

The *in vivo* study protocol, in accordance with the Guide for the Care and Use of Laboratory Animals that was established by the United States National Institutes of Health, was approved by the Animal Ethic Review Committees of the Second Military Medical University (Shanghai, China). After one week of acclimatization, the rats were randomly divided into four groups (each consists of 6 rats): (A) sham group (without coronary artery ligation), (B) MI group (with coronary artery ligation), (C) resina draconis-treated MI group (with coronary artery ligation), and (D) isosorbide dinitrate- (positive control drug)-treated MI group (with coronary artery ligation). Rats in group A and B received vehicle (CMC-Na), group C received total flavone extract of resina draconis (200 mg/kg/day), and group D received isosorbide dinitrate (5 mg/kg/day). Drugs and vehicle were orally administrated once per day for 5 consecutive days. Before the operation, rats were on overnight fast with free access to water. MI model was induced 0.5 h after gavage at day five by ligating left anterior descending coronary artery. Anterior thoracotomy was performed under sterile conditions to open the pericardium. The heart was then rapidly exteriorized. The 2-3 mm distal part was ligated from the left anterior descending coronary artery using a 6–0 polypropylene suture [18].

To confirm MI status and evaluate efficacies of the drugs, electrocardiograms (ECG) were recorded by MPA 2000 biosignal analysis system (Alcott Biotech Co. Ltd., Shanghai, China). Serum concentrations of LDH and CK were measured by a UV-1100 ultraviolet spectrophotometer (Beijing Rayleigh Analytical Instrument Corporation, Beijing, China), and the myocardial infarct size was determined by dissecting the left ventricles into slices and then identifying and weighing the infarcted tissues [18]. Blood samples were collected from femoral arteries, after which rats were sacrificed according to our *in vivo* study protocol. 1 mL of each blood sample was collected into the heparinized tube and centrifuged at 3500 g for 10 min, and then the plasma sample was separated and stored at −80°C until further analysis. 

### 2.3. Sample Preparation

Prior to UPLC-MS analysis, the plasma samples were thawed at room temperature. A volume of 400 *μ*L of methanol/ACN/acetone (1 : 1 : 1, v/v/v) was added to 100 *μ*L of plasma to precipitate the proteins. After vigorous shaking for 1 min and incubation on ice for 10 min, the mixture was centrifuged at 9560 g for 10 min at 4°C. The supernatant (350 *μ*L) was used for UPLC-MS analysis. To ensure the stability and repeatability of the UPLC-MS system, 20 *μ*L of each plasma sample was mixed to generate a pooled quality control (QC) sample, which was treated using the above method and analyzed together with the real samples.

### 2.4. UPLC-Q-TOF MS Analysis

Chromatography was performed on Agilent 1290 Infinity LC system. The column oven was set at 40°C. An acquity UPLC BEH C18 column (2.1 mm × 100 mm, 1.7 mm, Waters, Milford, MA, USA) was used. The mobile phase consisted of 0.1% formic acid (A) and ACN modified with 0.1% formic acid (B), using a gradient elution of 5% B at 0–2 min, 5%–95% B at 2–28 min, and 95% B at 28–30 min. The flow rate was 350 *μ*L min^−1^, and the injection volume was 4 *μ*L.

An Agilent 6538 Accurate-Mass Quadrupole Time-of-Flight (Q-TOF) mass spectrometer (Agilent, USA) was used in the study. The Q-TOF mass spectrometer was operated in both positive and negative ionization modes, with a capillary voltage of 4.0 kV, drying gas flow of 11 L min^−1^, and a gas temperature of 350°C. The nebulizer pressure was set at 45 psig. The fragmentor voltage was set at 120 V and skimmer voltage was set at 60 V. Data were collected in centroid mode and the mass range was set at *m/z* 100–1100 using extended dynamic range. The positive ionization mode, however, was found to give much more ions of the metabolites than the negative mode; we therefore used the data of the positive ionization mode in the following study. Potential biomarkers were analyzed by MS/MS. MS spectra were collected at 2 spectra/s, and MS/MS spectra were collected at 2 spectra/s, with a medium isolation window (~4 *m/z*) and a fixed collision energy of 10–40 V.

The typical sample sequence consisted of the consecutive analysis of one QC sample injection, followed by 5 real samples, then another QC sample injection, followed by another 5 real samples, and so forth. Samples were analyzed in a random order for a normal good practice.

### 2.5. Data Analysis and Metabolic Pathway Analysis

Raw data were converted to common data format (.mzData) files using a file converter program available in Agilent MassHunter Qualitative software, during which the isotope interferences were eliminated. Then the converted files were imported to the XCMS software (http://metlin.scripps.edu/xcms/) for nonlinear alignment in the time domain, automatic integration, and extraction of the peak intensities, with default parameter settings except that fwhm was 10, bw was 10, and snthersh was 5. The variables presented in at least 80% of the samples in each group were extracted. After that, a matrix containing the intensities of 1678 metabolite ions was normalized to the total area to correct for the MS response shift between injections due to any possible intra- and interday variations. Then the data were enrolled in the final data set for multivariate statistical analysis and pattern recognition analysis.

After mean centering and pareto scaling which increase the importance of low abundance ions without significant amplification of noise, the data were introduced to SIMCA-P V11.0 (Umetrics, Sweden) for partial least squares discriminant analysis (PLS-DA) and principal component analysis (PCA). The data show a normal distribution. *R* ^2^ and *Q* ^2^ which represent the goodness of the fitting and prediction ability of the models were used for evaluation of the models [19].

Potential metabolite markers were extracted using a statistically significant threshold of the variable importance in the projection (VIP) constructed from the PLS-DA analysis, where the markers were chosen based on their contributions to the variation and correlation within the data set, and two-tailed Student's *t*-test on the normalized raw data. The variables with VIP values larger than 1.5 and *P* values less than 0.05 were selected [19]. To facilitate observing and comparing the metabolic characteristics of various groups, heat maps exhibiting contributions of the significant variables in the corresponding groups were constructed using the MetaboAnalyst tool (http://www.metaboanalyst.ca/MetaboAnalyst/faces/Home.jsp/).

Metabolic pathway analysis was performed by MBRole [20] and ingenuity pathway analysis (IPA, http://www.ingenuity.com/, trial version) based on the database sources including KEGG (http://www.genome.jp/kegg/), Human Metabolome Database (http://www.hmdb.ca/), and PubChem (http://www.ncbi.nlm.nih.gov/pccompound/) to identify the affected metabolic pathways and facilitate further biological interpretation.

## 3. Results and Discussion

### 3.1. Evaluation of MI Model and Effects of Resina Draconis on MI Rats

**Figure 1:**
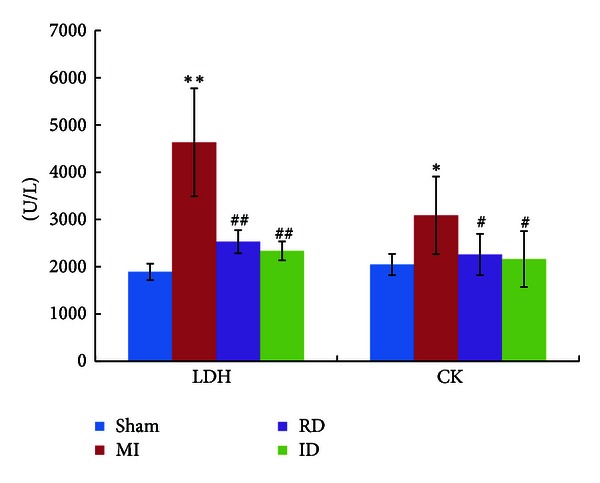
LDH and CK levels (U/L) in sham, MI, resina-draconis (RD-), and isosorbide-dinitrate- (ID-) treated MI groups. Data were expressed as mean ± SD. Significant comparison was based on two-tailed Student's *t*-test. *indicates *P* < 0.05, and **indicates *P* < 0.01 (with respect to the sham group); ^#^indicates *P* < 0.05, and ^##^indicates *P* < 0.01 (with respect to the MI group).

**Figure 2:**
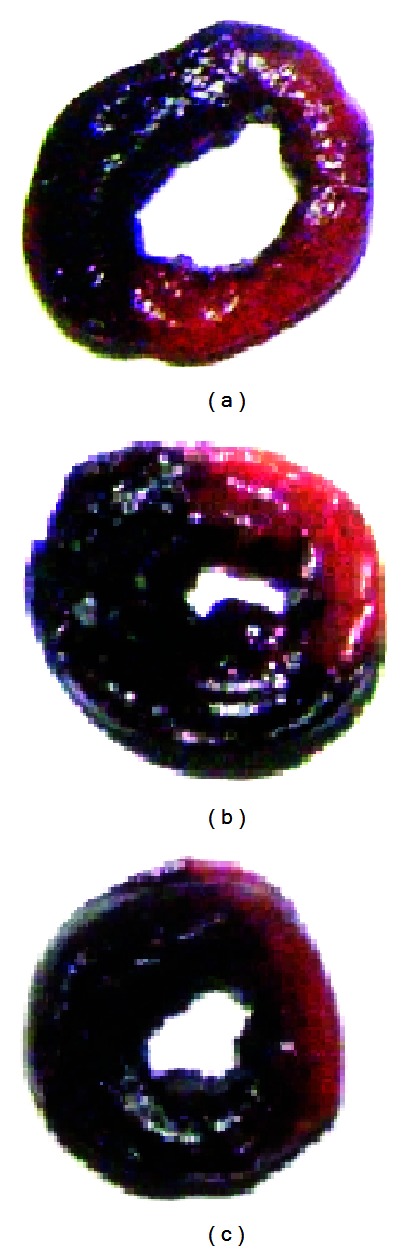
Typical photos of infarcted left ventricle slices of MI- (a), resina draconis- (b), and isosorbide dinitrate- (c) treated groups. The pale zones were the infarcted tissues.

Serum enzyme activities are important parameters for the evaluation of MI [21]. In this study, significant elevations of serum LDH (2.45 fold) and CK (1.51 fold) levels were observed in the MI group compared to those in the sham group, indicating that the MI rats suffered from myocardial ischemia caused by ligation of the coronary artery. After resina draconis/isosorbide dinitrate treatment, however, the increased serum enzyme levels were suppressed (Figure 1). In ECG the T-wave and ST-segment alterations are generally considered as a main index to evaluate MI status. In our study, altered T waves and ST segments were ameliorated by resina draconis or isosorbide dinitrate treatment to various degrees (See Figure S1 in Supplementary Material available online at http://dx.doi.org/10.1155/2013/438680). At the same time, percentage of the infarct size (ratio of infarcted heart weight to left ventricle weight) was significantly decreased in the resina draconis-treated group (24.0%  ±  5.96%) compared to the MI group (33.4%  ±  3.20%) (*P* = 0.0025) (Table S1).  Figure 2 displayed typical photos of infarcted left ventricles slices, where obviously the resina draconis/isosorbide dinitrate-treated groups had decreased infarct sizes comparing to the MI group. All these results demonstrate that resina draconis is effective for alleviating myocardial ischemia and protecting myocardial tissues. 

### 3.2. UPLC-MS Analysis

Reproducibility and stability of the UPLC-MS method were evaluated by calculating the relative standard deviations (RSDs) of the same ten peak areas from the pooled QC sample that was repetitively analyzed at the beginning, the end, and after every five injections through the analytical run. It was found that RSDs for the retention times and areas of these peaks in QC samples were 0.12%–0.35% and 3.08%–4.62%, respectively, demonstrating the robustness of our analytical method. Typical UPLC-MS TIC chromatograms of the samples from sham, MI, resina draconis, and isosorbide dinitrate-treated groups were shown in Figure S2.

### 3.3. Metabolic Profiling of MI Rats and Screening of the Metabolite Markers

A PLS-DA model was established based on the data of sham and MI groups, to evaluate the systemic changes in the metabolome of MI rats. Three latent variables (LV) were calculated, and the cumulative *R* ^2^ *Y* and *Q* ^2^ were 0.978 and 0.606, respectively. No overfitting of the data was observed based on the results of the permutation test (the *R* ^2^ *Y*-intercept was 0.443, and the *Q* ^2^-intercept was −0.021).

As shown in the score plot (Figure S3(a)), all the samples in these groups fell well inside the 95% confidence interval which was represented by an ellipse, and the sham and MI groups were clearly separated along the first LV, indicating that plasma biochemical perturbation significantly happened in the MI group. From the corresponding loading plot (Figure S3(b)), the variables (ions) far away from the origin contribute significantly to this model. To screen the statistically important variables (ions) related to MI, a strategy combining VIP and two-tailed Student's *t*-test was used. 69 variables with the VIP value larger than 1.5 and *P* value less than 0.05 were selected and considered as potential markers representing the metabolic characteristics of MI rats. A heat map (Figure S4) based on these potential markers was constructed using Ward's method, showing unsupervised hierarchical clustering of the sham and MI groups, which is consistent with the results in Figure S3(a).

Structure identification of the significant metabolites was performed according to their molecular ion mass and MS/MS product ion analysis compared with authentic standards or database resources. Here we take L-tryptophan (with the [M+H]^+^ quasi-molecular ion peak at *m/z* 205.0977) as an example to illustrate our metabolite identification strategy. According to its accurate mass, C_11_H_12_N_2_O_2_ was calculated as the most possible molecular formula by Agilent MassHunter software. Then, from its tandem MS spectra, three major fragment ions were found at *m/z* 188.07, 146.06, and 159.09, which represent the fragments of [C_11_H_12_N_2_O]^+^ (or [C_11_H_9_NO_2_]^+^), [C_9_H_8_NO]^+^, and [C_10_H_11_N_2_]^+^, respectively [22]. Then, the above information was searched and matched in databases of HMDB (http://www.hmdb.ca/), METLIN (http://metlin.scripps.edu/), and KEGG (http://www.kegg.jp/). Finally, this ion of *m/z* 205.0977 was validated by a standard compound and identified as L-tryptophan (Figure S5). A total of twenty metabolites were finally identified as MI markers and listed in Table 1.

### 3.4. Metabolic Pathway Analysis of MI

Based on the identified metabolite markers, a functional enrichment analysis facilitating further biological interpretation was performed using MBRole (18) to reveal the most relevant pathways involved in MI. It was shown in Table 2 that the revealed metabolite markers were mainly involved in the following pathways: aminoacyl-tRNA biosynthesis (L-phenylalanine, L-valine, L-tryptophan, L-tyrosine, and methionine), phenylalanine, tyrosine, and tryptophan biosynthesis (L-tyrosine, L-tryptophan, and L-phenylalanine), vascular smooth muscle contraction (cGMP, cAMP), melanogenesis (cAMP, L-Tyrosine), sphingolipid metabolism (sphinganine, phytosphingosine), phenylalanine metabolism (L-phenylalanine, L-tyrosine), ABC transporters (L-valine, L-phenylalanine), purine metabolism, and gap junction (cGMP, cAMP).

Combining the results in Tables 1 and 2, it is likely that MI perturbed several amino acid pathways, including phenylalanine metabolism and phenylalanine, tyrosine, and tryptophan biosynthesis. In the MI group, phenylalanine level decreased whereas tyrosine was upregulated, possibly due to an increased activity of phenylalanine hydroxylase (PAH) that catalyzes the conversion of phenylalanine to tyrosine in phenylalanine metabolism [23, 24]. The breakdown of tryptophan was consistent with previous reports [25, 26]. Tan et al. reported accumulation of two metabolites of tryptophan metabolism, N-methylnicotinamide and indole-3-acetamide in MI rats [26], suggesting an increased clearance of tryptophan through the tryptamine pathway. 

Cyclic AMP (cAMP) and cyclic GMP (cGMP), the main messengers that mediate the vascular smooth muscle contraction pathway [27], were upregulated in MI group. cAMP and cGMP both regulate the slow Ca^2+^ channels that allow Ca^2+^ influx into the myocardial cells, but cAMP (stimulating) and cGMP (inhibiting) have opposite effects [28]. During cardiac ischemia the increase in cAMP concentration is the cause of ventricular fibrillation [29], which would exacerbate ischemia-reperfusion injury by elevating cytosolic calcium levels [30]. The increased synthesis of cGMP is probably related to activation of NO synthases and production of NO in the ischemic myocardium [29, 31], which was proven to counter cAMP-induced increase in the slow inward Ca^2+^ current [32, 33]. As a result, MI may impair regulation of the vascular tone, which is associated with the pathophysiology of vascular diseases. 

MI may also activate the sphingolipid metabolism. Sphingolipids are highly bioactive compounds and are involved in diverse cell processes, including cell-cell interaction, cell proliferation, cell differentiation, and apoptosis [34]. Emerging evidence has shown that sphingolipid-mediated signal transduction pathways play a significant role in cardiovascular pathophysiology [35]. In our study, three phospholipids, including phytosphingosine, sphinganine, and hexadecasphinganine, had increased levels in MI group, showing the activated sphingolipid metabolism.

Besides, lysophosphatidylcholines (LPC) is formed by hydrolysis of phosphatidylcholines (PC) by the enzyme phospholipase A2. Choy et al. reported a 51% reduction in the biosynthesis of PC in the ischemic heart and attributed this to a severe decrease in ATP level which resulted in a diminished conversion of choline into phosphocholine [36]. In our study, levels of quite a few LPC and PC were altered in the MI group, also indicating the disturbed phospholipid catabolism. 

### 3.5. Metabolomic Evaluation of Resina Draconis Treatment on MI Rats

As shown in Section 3.1, resina draconis has shown positive effects on MI rats for reducing the serum LDH and CK levels, ameliorating alterations in ECG, as well as decreasing the heart infarct sizes. To further unveil the precise mechanisms of resina draconis on MI, the metabolomics approach was employed for a second time to determine the metabolic profiles of MI rats treated with resina draconis and then analyze the metabolic networks and involved pathways. 

**Figure 3:**
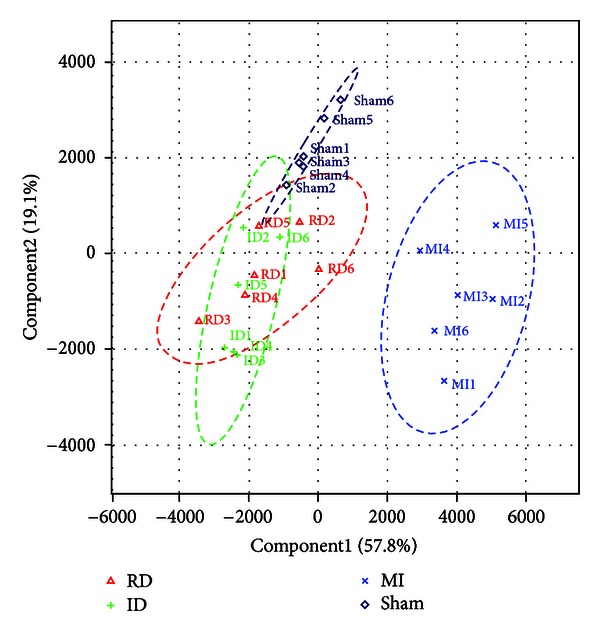
PCA scores plot based on data of sham (navy blue diamonds), MI (blue crosses), resina draconis (RD, red triangles), and isosorbide dinitrate (ID, green crosses) groups.

An unsupervised PCA model was established based on the levels of the metabolite markers in all the groups. The first two principal components explained 57.8% and 19.1% of the variance in the data, respectively. According to the scores plot of this model (Figure 3), both the resina draconis- and isosorbide dinitrate-treated groups were close to the sham group, and all these three groups were located in the left side of the figure, which were clearly separated from the MI group on the right side. This was quite consistent with the histopathology results in Section 3.1.

**Figure 4:**
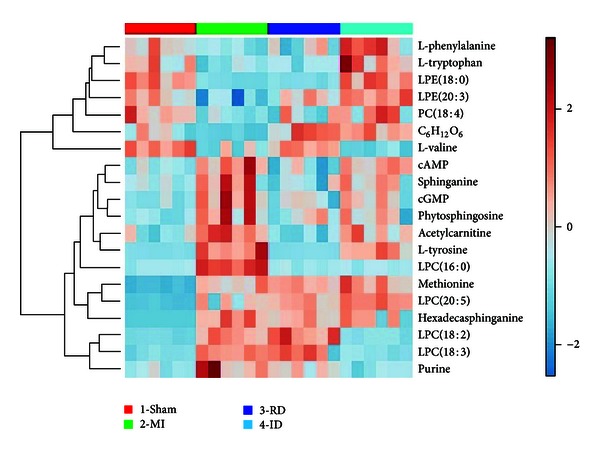
Heat map visualization based on the levels of potential biomarkers in MI, sham, resina-draconis (RD-), and isosorbide-dinitrate (ID-) treated groups. Columns correspond to samples, and variables marked on the right correspond to metabolites. Color key indicates metabolite expression value, red: highest, blue: lowest.

According to Table 1 and the heat map based on the level changes of potential biomarkers in all the groups (Figure 4), it could be concluded that trends of a number of metabolite markers were respectively reversed by resina draconis or isosorbide dinitrate administration. Specifically, levels of phytosphingosine, sphinganine, acetylcarnitine, cGMP, cAMP, L-tyrosine, and L-valine were significantly reversed after resina draconis treatment, whereas isosorbide dinitrate treatment significantly reversed the levels of LPC(18:3), LPC(18:2), purine, PC(18:4), LPE(18:0), L-phenylalanine, and L-tryptophan. Both drugs reversed the levels of LPC(16:0), LPE(20:3), and C_6_H_12_O_6_, yet they failed to alter the trends of hexadecasphinganine, LPC(20:5), and methionine. 

**Figure 5:**
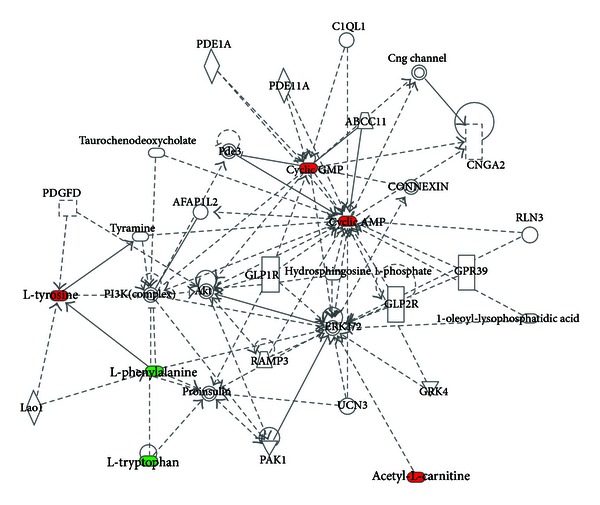
Molecular network based on some key metabolites reversed by resina draconis or isosorbide dinitrate treatment. Metabolites were represented as nodes, and the biological relationship between two nodes is represented as a line. The colored nodes represent metabolites that occur in our data, while the transparent entries are molecules from the Ingenuity Knowledge Database. Red nodes represent upregulated metabolites, and green nodes represent downregulated metabolites. Solid lines between molecules indicate a direct physical relationship between molecules, and dotted lines indicate indirect functional relationships.

Then, to further understand the correlation between these metabolites, bioinformatics analysis based on IPA was performed leading to the identification of biological association networks and delineating a global view of interactions of some key metabolites reversed by resina draconis or isosorbide dinitrate treatment (Figure 5).

Synthesizing the obtained information, it was indicated that, firstly, it is known that vasodilators can cause relaxation of vascular smooth muscle by activating cAMP phosphodiesterases which reduces cAMP and Ca^2+^ levels [37]; resina draconis appeared to more significantly intervene vascular smooth muscle contraction than isosorbide dinitrate, one of the known vasodilators, since resina draconis more greatly downregulated cAMP and cGMP levels. Secondly, resina draconis could restore the sphingolipid metabolism. It has been revealed that some key enzymes associated with sphingolipid metabolism, for example, sphingomyelinases (SMases) that hydrolyze sphingomyelin to create a cascade of bioactive lipids including sphinganine and phytosphingosine, were upregulated in myocardial ischemia [38]. Hence, resina draconis may be involved in these enzymes since it significantly downregulated phytosphingosine and sphinganine levels of MI rats. Thirdly, resina draconis significantly downregulated L-tyrosine whereas isosorbide dinitrate failed. We hence supposed that PAH, which catalyzes the conversion of phenylalanine to tyrosine, may be one possible target of resina draconis in restoring the perturbed metabolic network of MI rats. As to other metabolites influenced by resina draconis, L-valine is one of the branched chain essential amino acids (BCAAs) that are particularly involved in energy and muscle metabolism. In MI, BCAAs derived from the mobilization of muscle protein may be important alternative energy substrates for the heart [39], after which L-valine could be metabolized to carbohydrates as a compensation of energy. The accumulation of acetylcarnitine in MI group may be related to the increase of acetyl-CoA so that it can facilitate movement of acetyl-CoA into the matrices of mitochondria during the oxidation of fatty acids [40]. Hence, the buildup of L-valine and decrease of acetylcarnitine in resina draconis-treated group indicated the restoration of these disturbed pathways.

Four out of seven of the metabolites influenced by isosorbide dinitrate, LPC(18:3), LPC(18:2), PC(18:4), and LPE(18:0) were involved in the phospholipid catabolism, which implied that isosorbide dinitrate mostly intervened the phospholipid catabolism of MI rats. Both L-phenylalanine and L-tryptophan were significantly upregulated by isosorbide dinitrate treatment, indicating the partial restore of phenylalanine, tyrosine, and tryptophan biosynthesis pathway.

**Figure 6:**
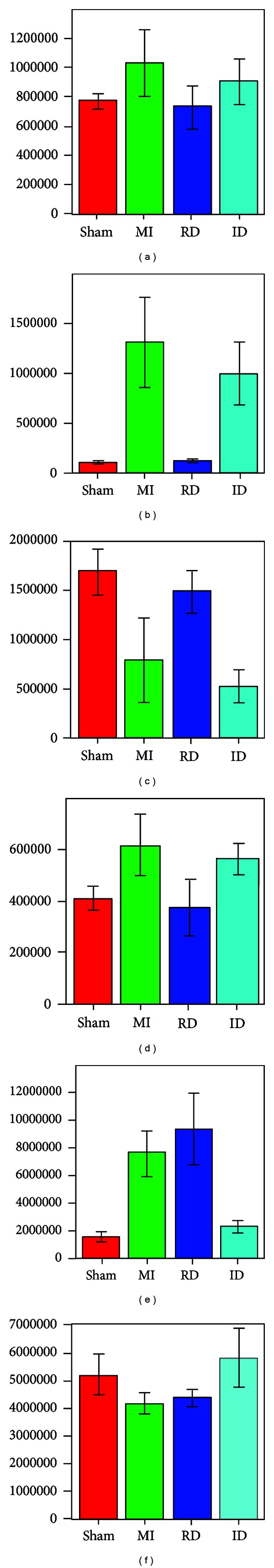
Levels of six representative metabolites in various pathways in sham, MI, resina-draconis- (RD-), and isosorbide-dinitrate- (ID-) treated groups. (a) Sphinganine (in sphingolipid metabolism); (b) L-tyrosine (in phenylalanine metabolism); (c) L-valine (in BCAA metabolism); (d) cAMP (in vascular smooth muscle contraction); (e) LPC(18:2) (in phospholipids metabolism); (f) L-tryptophan (in phenylalanine, tyrosine, and tryptophan biosynthesis).

In all, according to our results, resina draconis seems to adjust the disturbed vascular smooth muscle contraction, sphingolipid metabolism, phenylalanine metabolism, and BCAA metabolism to alleviate MI. Levels of six representative metabolites in sphingolipid metabolism, phenylalanine metabolism, BCAA metabolism, vascular smooth muscle contraction, phospholipids metabolism, and phenylalanine, tyrosine, and tryptophan biosynthesis in all the groups were shown in Figure 6.

## 4. Conclusions

In this study, an UPLC/MS-based metabolomics approach was used to characterize the metabolic profiles of MI and investigate the mechanisms of resina draconis on MI rats. From our results, a number of metabolite markers in MI rats were reversed by resina draconis, from which some possible metabolic pathways related to resina draconis treatment were revealed. It is likely that resina draconis alleviates MI through regulating the disturbed vascular smooth muscle contraction, sphingolipid metabolism, phenylalanine metabolism, and BCAA metabolism. These findings further extended our understanding on the metabolic changes related to MI and may provide new clues for exploring the mechanisms of resina draconis on MI.

## Supplementary Material

In our study, altered T waves and ST segments were ameliorated by resina draconis or isosorbide dinitrate treatment to various degrees.

## Figures and Tables

**Table 1:** Identified plasma metabolite markers of MI rats.

No	Retention time (min)	*m*/*z*	Exact mass	ID	Identified results	MI versus sham*	RD versus MI^#^	ID versus MI^Δ^	Pathway
1	1.23	182.0813	181.0739	C00082	L-tyrosine	↑	↓		Phenylalanine, tyrosine, and tryptophan biosynthesis
2	10.20	318.3007	317.2930	C12144	Phytosphingosine	↑	↓		Sphingolipid metabolism
3	11.38	302.3057	301.2981	C00836	Sphinganine	↑	↓		Sphingolipid metabolism
4	1.18	204.1233	203.1158	C02571	Acetylcarnitine	↑	↓		Fatty acid transportation
5	11.45	346.3317	345.0474	C00942	Cyclic GMP	↑	↓		Purine metabolism
6	12.64	330.3367	329.0525	C00575	Cyclic AMP	↑	↓		Purine metabolism
7	1.17	118.0866	117.0790	C00183	L-valine	↓	↑		Valine, leucine and isoleucine biosynthesis
8	12.76	520.3408	519.3325	C04230, HMDB10386	LPC(18:2)	↑		↓	Phospholipids metabolism
9	13.28	518.3227	517.3168	HMDB10388	LPC(18:3)	↑		↓	Phospholipids metabolism
10	18.99	121.0509	120.0436	HMDB01366	Purine	↑		↓	Purine metabolism
11	14.81	482.3243	481.3173	HMDB11130	LPE(18:0)	↓		↑	Phospholipids metabolism
12	17.91	780.5538	779.5465	C00157	PC(18:4)	↓		↑	Phospholipids metabolism
13	2.22	166.0866	165.0790	C00079	L-phenylalanine	↓		↑	Phenylalanine, tyrosine, and tryptophan biosynthesis
14	4.16	205.0977	204.0965	C00078	L-tryptophan	↓		↑	Phenylalanine, tyrosine, and tryptophan biosynthesis
15	13.27	496.3415	495.3325	C04230	LPC(16:0)	↑	↓	↓	Phospholipids metabolism
16	14.81	504.3063	503.3012	HMDB11516	LPE(20:3)	↓	↑	↑	Phospholipids metabolism
17	1.35	203.053	180.0634	NA	C_6_H_12_O_6_	↓	↑	↑	Unknown
18	10.28	274.2747	273.2668	C13915	Hexadecasphinganine	↑	↑	↑	Sphingolipid metabolism
19	12.77	542.3228	541.3168	C04230, HMDB10397	LPC(20:5)	↑	↑	↑	Phospholipids metabolism
20	1.18	150.0584	149.0511	C00073	Methionine	↑	↑	↑	Aminoacyl-tRNA biosynthesis

*↑ or ↓ represents the up- or downregulation of the metabolites in MI group compared with the sham group; ^#^↑ or ↓ represents the significant up- or downregulation of the metabolites in resina-draconis (RD-) treated group compared with the MI group; ^Δ^↑ or ↓ represents the significant up- or down-regulation of the metabolites in isosorbide dinitrate- (ID, positive control drug) treated group compared with the MI group.

**Table 2:** Metabolic pathway enrichment analysis of the metabolites identified as potential biomarkers.

Pathway name	*P* value	Adj. *P* value	Hits	Metabolites
Aminoacyl-tRNA biosynthesis	8.91*E* − 07	3.56*E* − 05	5	L-phenylalanine, L-valine L-tryptophan, L-tyrosine, and methionine
Phenylalanine, tyrosine, and tryptophan biosynthesis	4.93*E* − 05	9.86*E* − 04	3	L-tyrosine, L-tryptophan, and L-phenylalanine
Vascular smooth muscle contraction	0.002215	0.011075	2	cGMP, cAMP
Melanogenesis	1.14*E* − 04	0.00114	2	cAMP, L-tyrosine
Sphingolipid metabolism	0.002215	0.011075	2	Sphinganine, phytosphingosine
Phenylalanine metabolism	0.007401	0.032893	2	L-phenylalanine, L-tyrosine
ABC transporters	0.026777	0.101461	2	L-valine, L-phenylalanine
Purine metabolism	0.027902	0.101461	2	cAMP, cGMP
Gap junction	4.15*E* − 04	0.003319	2	cGMP, cAMP

*P* value, statistically assessed against the background set; adj. *P* value, *P* value corrected using the false discovery rate.
