# Supplementary material for: Metabolomics Study of Resina Draconis on Myocardial Ischemia Rats Using Ultraperformance Liquid Chromatography/Quadrupole Time-of-Flight Mass Spectrometry Combined with Pattern Recognition Methods and Metabolic Pathway Analysis
Source: Evid Based Complement Alternat Med. 2013 May 26;2013:438680. doi: 10.1155/2013/438680 (PMC3677627; doi:10.1155/2013/438680)
Supplement: Supplementary file 1 — In our study, altered T waves and ST segments were ameliorated by resina draconis or isosorbide dinitrate treatment to various degrees. [file 438680.f1.doc]

**Supplementary materials**

Metabolomics study of resina draconis on myocardial ischemia rats using ultra-performance liquid chromatography/quadrupole time-of-flight mass spectrometry combined with pattern recognition methods and metabolic pathway analysis

**Contents**

1. **FIGURE S1:** Electrocardiogram (ECG) of sham, MI, resina draconis and isosorbide dinitrate-treated MI rats.
2. **FIGURE S2:** Typical UPLC-MS TIC chromatograms of the plasma samples from the sham, MI, resina draconis and isosorbide dinitrate-treated MI groups.
3. **FIGURE S3:** Score and loading plots of the PLS-DA model based on data of sham and MI groups.
4. **FIGURE S4:** Heat map visualization based on the differential metabolites of importance for MI and sham groups.
5. **FIGURE S5:** Identification of a significant marker ([M+H]^+^ m/z 205.0977).
6. **TABLE S1:** Comparison of the infarcted sizes in sham, MI, resina draconis, and isosorbide dinitrate-treated groups.


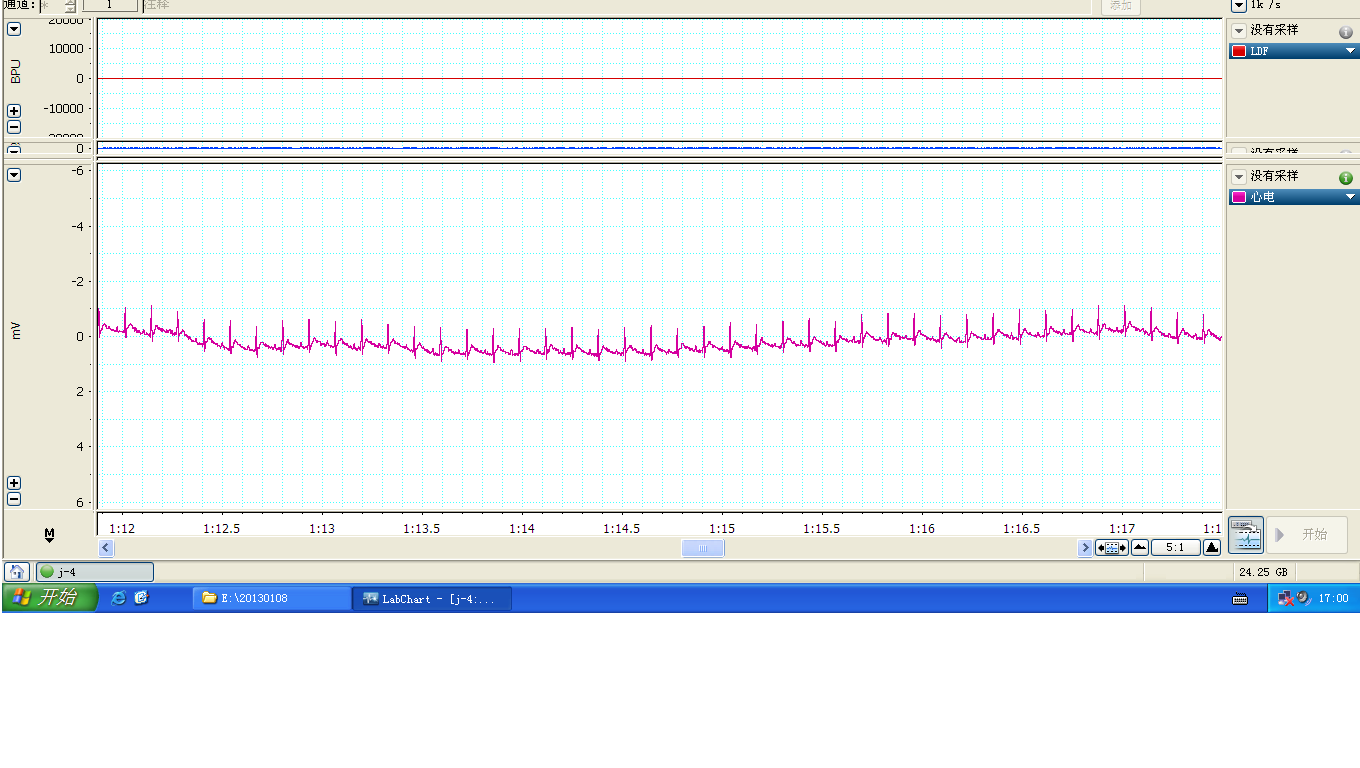


(a)


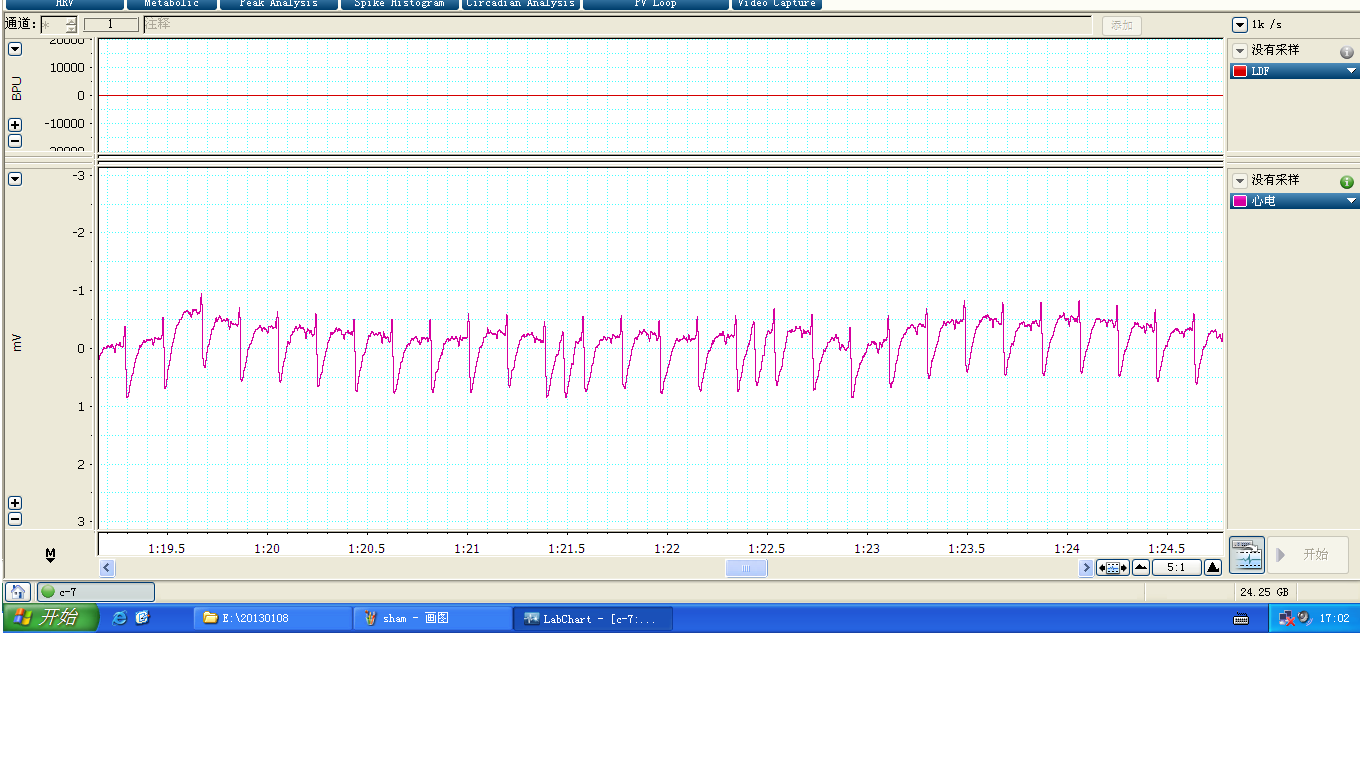


(b)


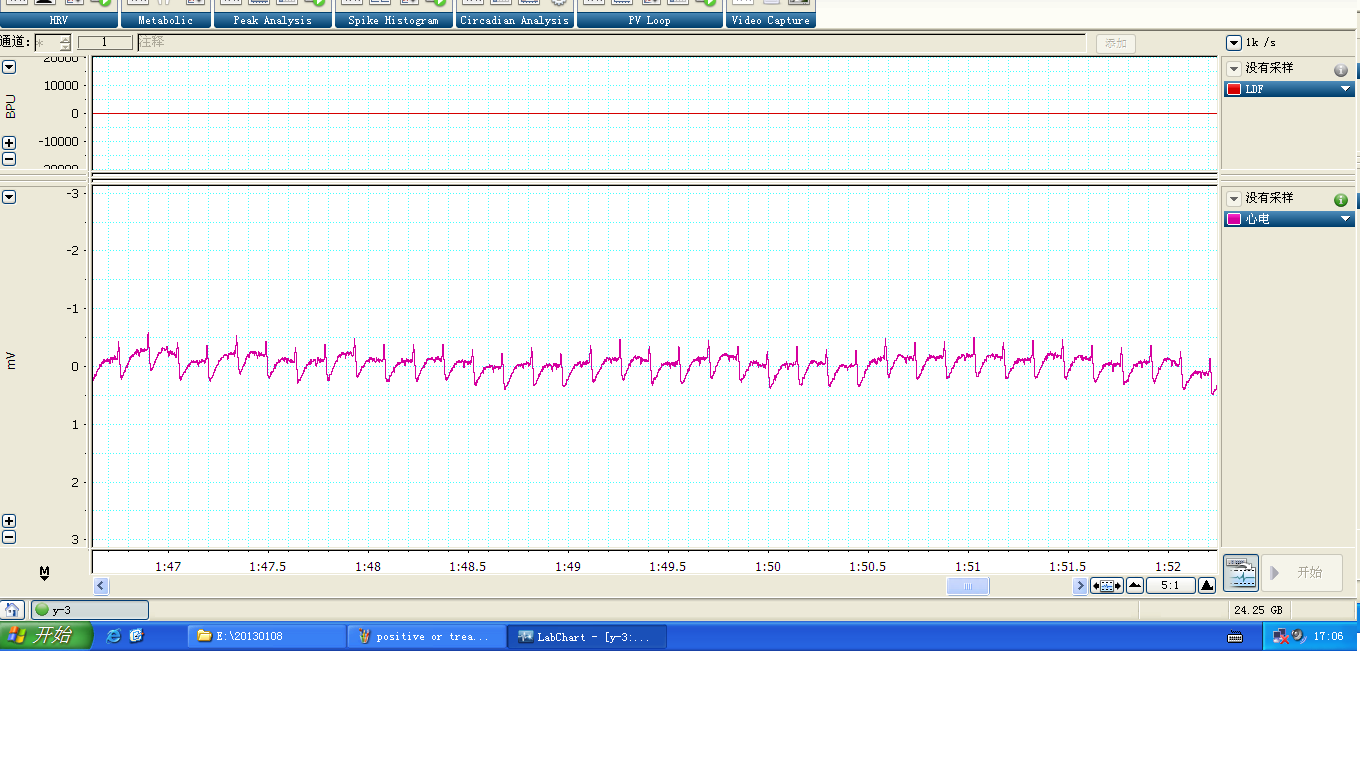


(c)


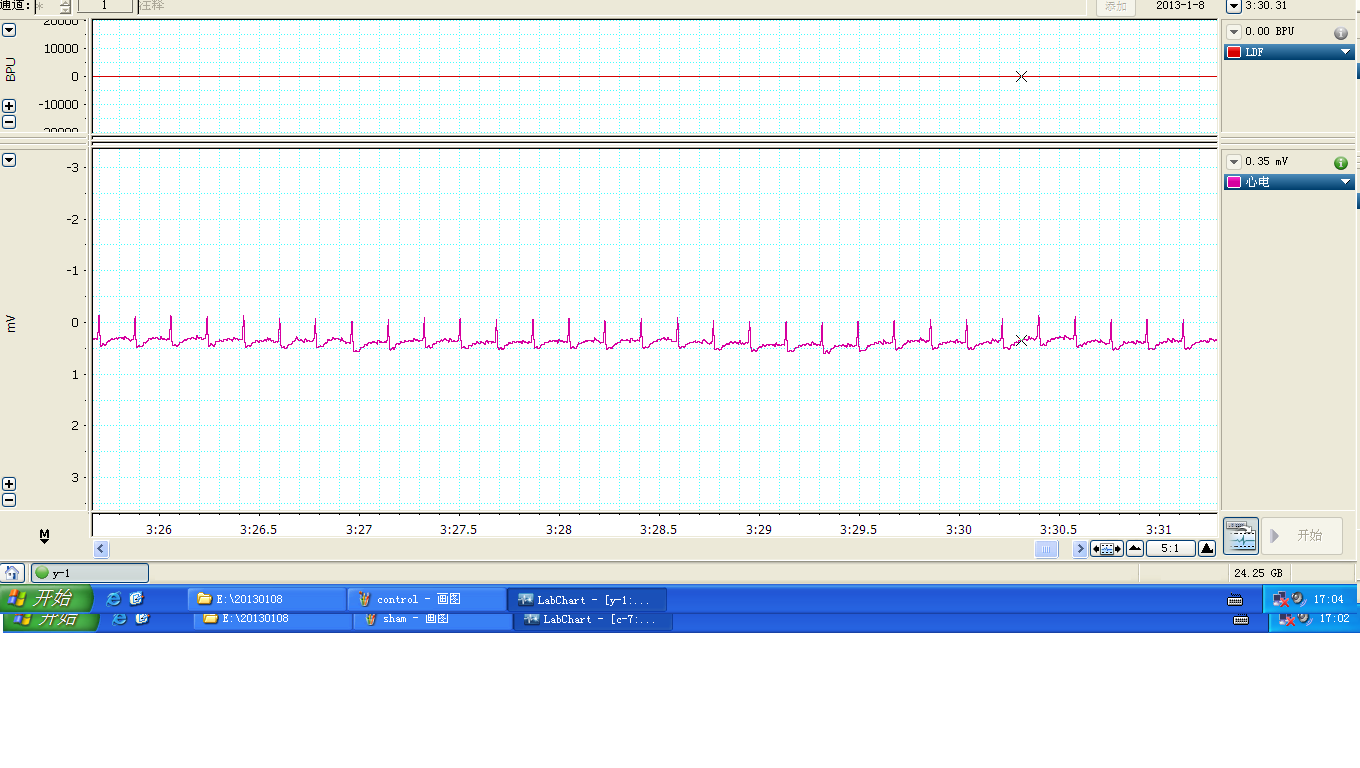


(d)

ST T wave

**FIGURE S1:** Electrocardiogram (ECG) of sham (a), MI (b), resina draconis (c) and isosorbide dinitrate (d)-treated MI rats.


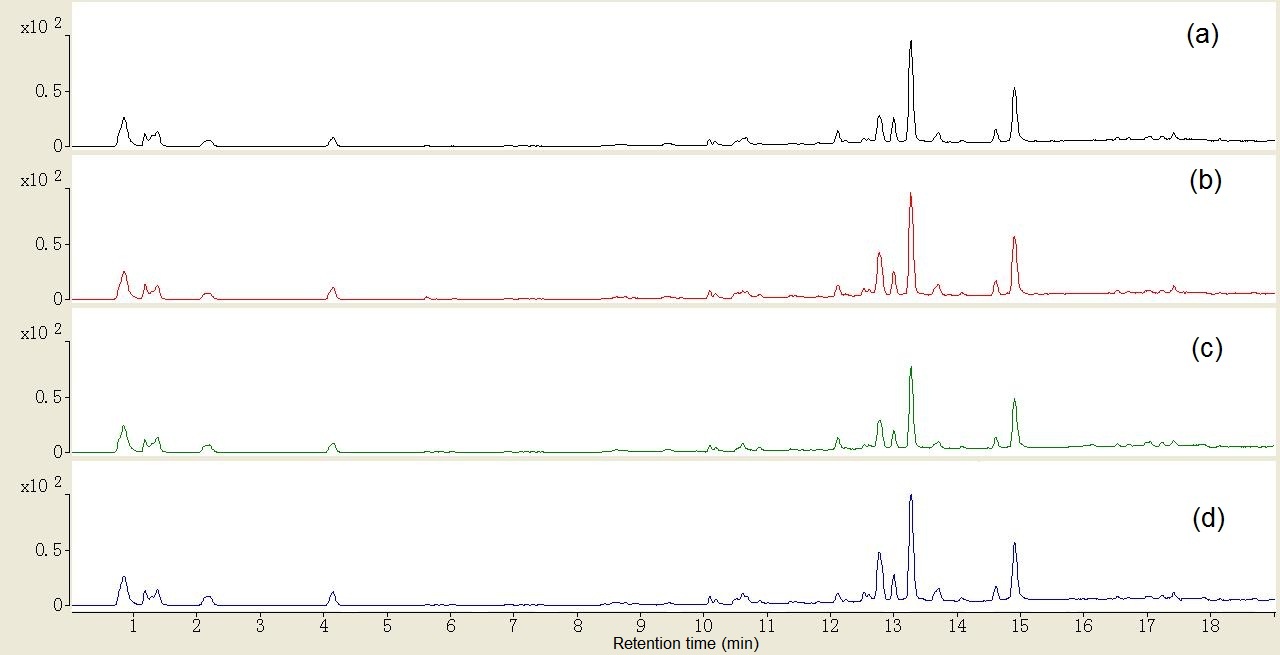


**FIGURE S2:** Typical UPLC-MS TIC chromatograms of the plasma samples from the four groups: (a) sham group; (b) MI group; (c) resina draconis-treated MI group; (d) isosorbide dinitrate-treated MI group.


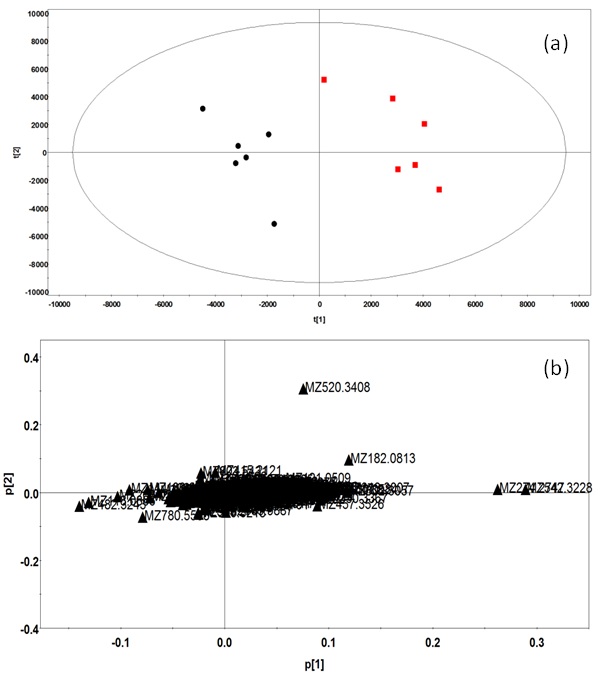


**FIGURE S3:** (a) Scores plot of the PLS-DA model based on data of sham (black circles) and MI groups (red squares); (b) Loadings plot of this model, the triangles indicate the variables (ions), and those far away from the origin contribute significantly to this model.


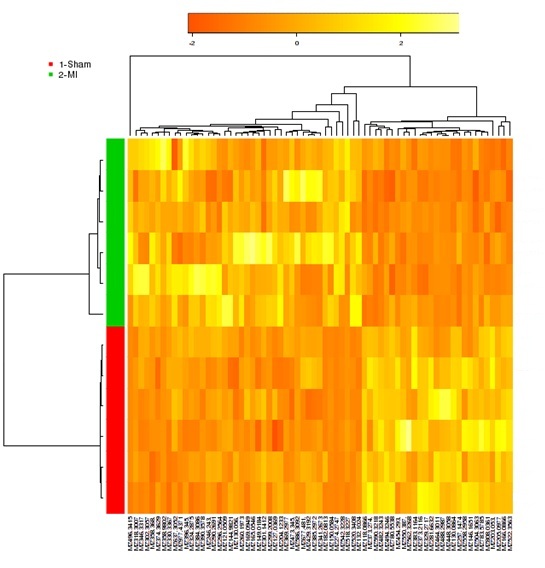


**FIGURE S4:** Heat map visualization based on the differential metabolites of importance for MI and sham groups. Rows correspond to samples, and variables marked on the bottom correspond to metabolites. Color key indicates metabolite expression value, red: lowest, yellow: highest.


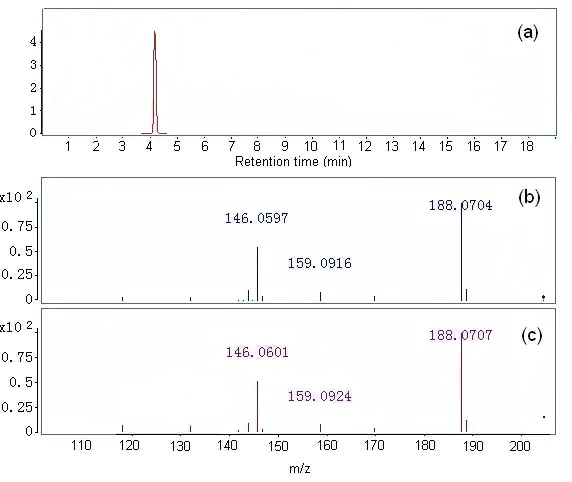


**FIGURE S5:** Identification of a significant marker ([M+H]^+^ m/z 205.0977). (a) Extracted ion chromatogram (EIC) of m/z 205.0977 (Rt = 4.16 min); (b) MS/MS spectrum of the ion; (c) MS/MS spectrum of a commercial standard L-tryptophan.

**TABLE S1:** Comparison of the infarcted sizes in sham, MI, resina draconis, and isosorbide dinitrate-treated groups.

| Groups | Infarcted weight  /left ventricle weight (%) | *p*-values^b^ |
| --- | --- | --- |
| Sham | NA^a^ |  |
| MI | 33.4±3.20 |  |
| Resina draconis treatment | 24.0±5.96 | 2.50E-03 |
| Isosorbide dinitrate treatment | 19.1±4.65 | 7.10E-06 |

^a^No infarcted area appeared in the sham group. ^b^ Significant difference compared with MI group.
